# Recent insights about pyrrolidine core skeletons in pharmacology

**DOI:** 10.3389/fphar.2023.1239658

**Published:** 2023-09-06

**Authors:** Samet Poyraz, H. Ali Döndaş, Naciye Yaktubay Döndaş, José M. Sansano

**Affiliations:** ^1^ Department of Basic Pharmaceutical Sciences, Faculty of Pharmacy, Çukurova University, Adana, Türkiye; ^2^ Department of Biotechnology, Institute of Natural and Applied Sciences, Çukurova University, Adana, Türkiye; ^3^ Department of Pharmacology, Faculty of Medicine, Çukurova University, Adana, Türkiye; ^4^ Department of Organic Chemistry, Centro de Innovación en Química Avanzada (ORFEO-CINQA), Instituto de Síntesis Orgánica (ISO), University of Alicante, Alicante, Spain

**Keywords:** pyrrolidine, pyrrolidine 2,5-dione, natural products, cytotoxicity, pharmaceuticals

## Abstract

To overcome numerous health disorders, heterocyclic structures of synthetic or natural origin are utilized, and notably, the emergence of various side effects of existing drugs used for treatment or the resistance of disease-causing microorganisms renders drugs ineffective. Therefore, the discovery of potential therapeutic agents that utilize different modes of action is of utmost significance to circumvent these constraints. Pyrrolidines, pyrrolidine-alkaloids, and pyrrolidine-based hybrid molecules are present in many natural products and pharmacologically important agents. Their key roles in pharmacotherapy make them a versatile scaffold for designing and developing novel biologically active compounds and drug candidates. This review aims to provide an overview of recent advancements (especially during 2015–2023) in the exploration of pyrrolidine derivatives, emphasizing their significance as fundamental components of the skeletal structure. In contrast to previous reviews that have predominantly focused on a singular biological activity associated with these molecules, this review consolidates findings from various investigations encompassing a wide range of important activities (antimicrobial, antiviral, anticancer, anti-inflammatory, anticonvulsant, cholinesterase inhibition, and carbonic anhydrase inhibition) exhibited by pyrrolidine derivatives. This study is also anticipated to serve as a valuable resource for drug research and development endeavors, offering significant insights and guidance.

## Graphical Abstract

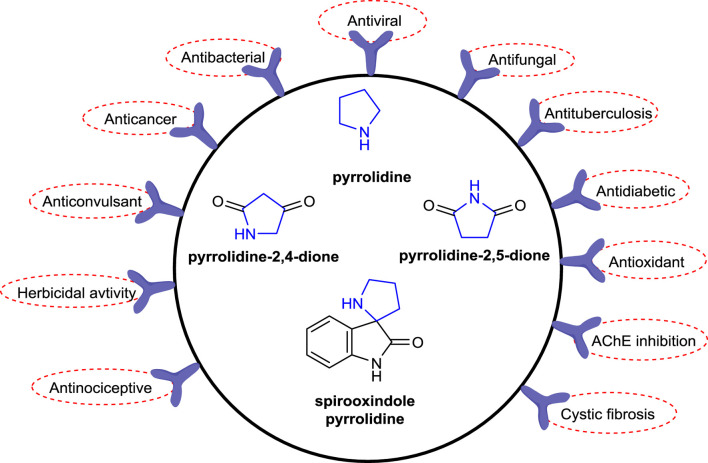

## 1 Introduction

**FIGURE 1:**
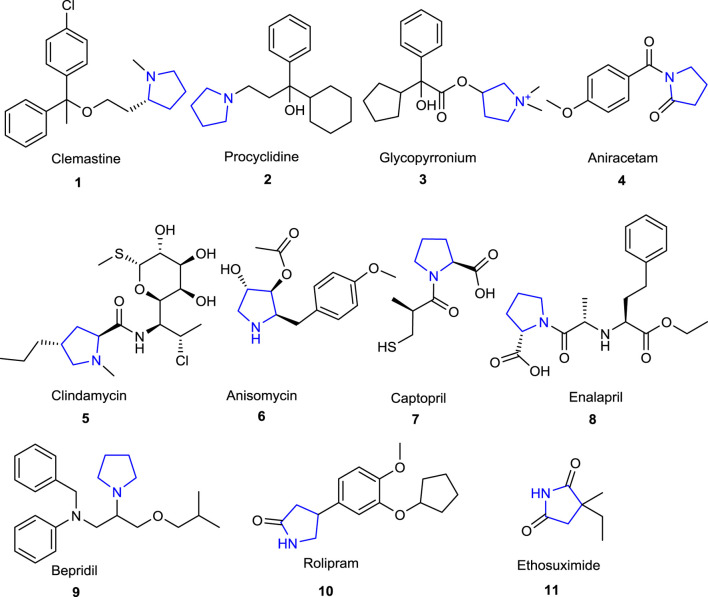
Pyrrolidine-based marketed drugs.

**FIGURE 2:**
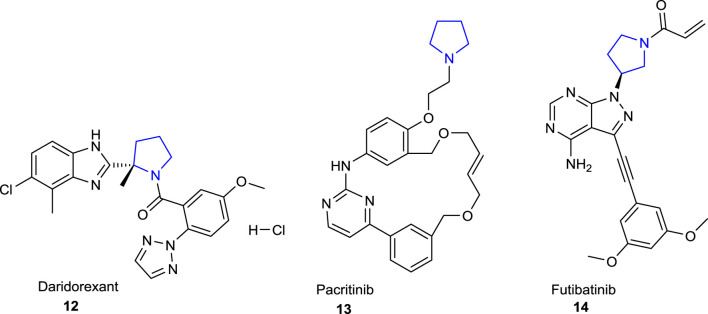
FDA-approved pyrrolidine-containing drugs in 2022.

The pyrrolidine ring, also known as tetrahydropyrrole, which is one of the important heterocyclic compounds containing five-membered nitrogen atoms, is the core structure of numerous biologically and pharmacologically active molecules, as well as many alkaloids and bioactive compounds (Bhat and Tilve, 2014; Li Petri et al., 2021). Compounds bearing pyrrolidine scaffolds continue to be utilized as intermediates in drug research and development (R&D) studies for the development of molecules that could be new drug candidates (Jeelan Basha et al., 2022; Bhat et al., 2023a). Some pyrrolidine derivatives are known to be employed as pharmacophore groups, with some having antibacterial (Huang et al., 2015), antifungal (Lukowska-Chojnacka et al., 2019), antiviral (Moni et al., 2015), antimalarial (Meyers et al., 2019), antitumoral (Li et al., 2020), anti-inflammatory (Jan et al., 2020), anticonvulsant (Góra et al., 2020), and antioxidant (Hussain et al., 2019) activities, while others have diverse enzyme inhibitory effects (Zhou et al., 2019; Salve and Jadhav, 2021; Toumi et al., 2021; Poyraz et al., 2023a). In addition, several compounds with essential bioactive characteristics have been reported in the literature that comprise fused or spiropyrrolidine rings (Łowicki and Przybylski, 2022). The well-known drugs with a pyrrolidine ring in their structural skeleton (Figure 1) include clemastine **1** (antihistaminic), procyclidine **2**, glycopyrronium **3** (anticholinergic), aniracetam **4** (anti-Alzheimer), clindamycin **5**, anisomycin **6** (antibacterial), captopril **7**, enalapril **8**, bepridil **9** (antihypertensive), rolipram **10** (antidepressant), and ethosuximide **11** (antiepileptic) (https://go.drugbank.com/categories/DBCAT000663). Furthermore, the therapeutic molecules with a pyrrolidine ring in their structures such as daridorexant **12** (insomnia), pacritinib **13** (JAK-2 inhibitor), and futibatinib **14** (FGFR-4 inhibitor) were approved by the FDA in 2022 (Benedetto Tiz et al., 2022) (Figure 2).

The molecular diversity and complexity of such pyrrolidine-based molecules allow us to design and develop more active and less toxic drug candidates by taking into account the elucidation of structure–activity relationship (SAR) and quantitative structure–activity relationship (QSAR) in the synthetic pathway (Vitaku et al., 2014; Bhat et al., 2023a; Bhat et al., 2023b). So, in this review, the most relevant pharmaceutical uses of pyrrolidine frameworks are detailed.

## 2 Polysubstituted pyrrolidines

Our research group designed the synthesis of significant hybrid compound series containing cyclic or bicyclic pyrrolidine rings and reported their bioactive properties. The pyrrolidine hybrids were incorporated with important pharmacophore moieties such as *N*-benzoylthiourea, thiohydantoin, thiazole, imidazole, and indole (Belveren et al., 2017; Poyraz et al., 2017; Poyraz et al., 2018; Belveren et al., 2019; Poyraz et al., 2023b).

Recently, our research group designed *N-*benzoylthiourea-pyrrolidine carboxylic acid derivative compounds carrying a series of imidazole rings as cholinesterase inhibitors, and their acetylcholinesterase (AChE) and Butyrylcholinesterase (BuChE) inhibition properties were compared with those of the reference tacrine. The most active compounds were found to be phenyl- and methyl-substituted **15g** (IC_50_: 0.029 µM), **15h** (IC_50_: 0.041 µM), and **16g** (IC_50_: 0.087 µM) with an indole ring in their structures (Poyraz et al., 2023a). Moreover, as part of our ongoing research work related to pyrroline-based potential bioactive molecules, we recently reported the design and synthesis of novel pyrrolidine-based benzenesulfonamide derivatives as carbonic anhydrase/acetylcholinesterase inhibitors, together with their antimicrobial properties. Thus, compounds **19a** and **19b** bearing 2,4-dimethoxyphenyl and 4-methoxyphenyl substituents were the most promising AChE inhibitor candidates with the *K*i values of 22.34 ± 4.53 nM and 27.21 ± 3.96 nM, respectively, compared to tacrine inhibition. Compound **18** without a sulfonamide moiety showed significant inhibition of the hCAI enzyme, with a *K*i value of 17.61 ± 3.58 nM, approximately 10 times greater than that of the reference AZA (164.22 ± 14.13 nM), and for the hCAII enzyme, with a *K*i value of 5.14 ± 0.61 nM, approximately 26 times greater than that of the reference AZA (132.53 ± 7.44 nM) (Poyraz et al., 2023a).


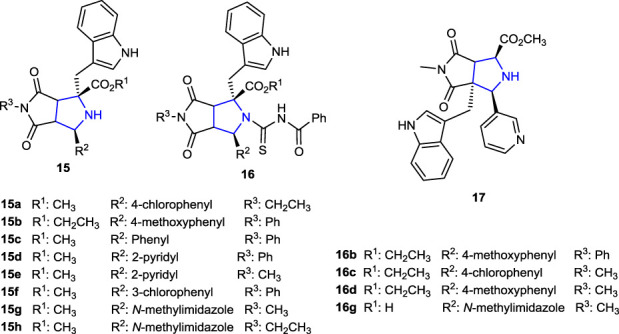



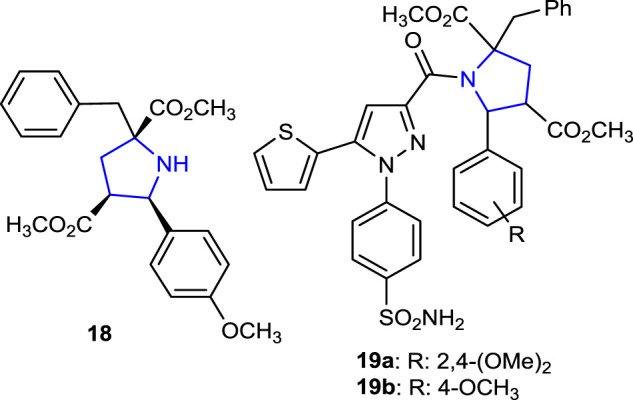


The pyrrolidine core is part of the general polycyclic framework **20**, a potential bioactive compound for the treatment of cystic fibrosis (CF). In this sense, more sophisticated analogs **21** containing this polycycle **20** can be classified as promising drugs for this purpose. Some studies regarding herbicide and pesticide activities, as well as surveys for plant growth control, are under investigation (Belabbes et al., 2023).


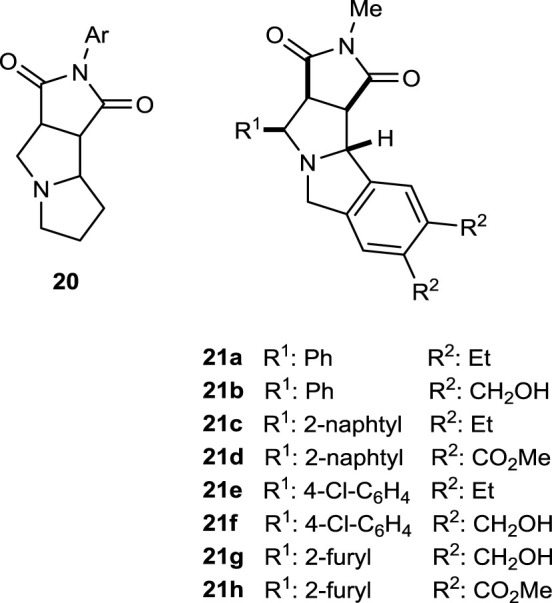


Frejat et al. (2022) synthesized 1,2,4-oxadiazole pyrrolidine derivatives and tested their antibacterial activity against different bacterial strains by inhibiting DNA gyrase and topoisomerase IV which are important targets for antibacterial agents. Compounds **22a** (180 ± 20 nM), **22b** (210 ± 20 nM), **22c** (120 ± 10 nM), **22d** (250 ± 20 nM), and **22e** (270 ± 20 nM) inhibited *E. coli* DNA gyrase at concentrations similar to the standard novobiocin (IC_50_ = 170 nM). Notably, the 4-chlorophenyl group is present in the structures of three (**22b–d**) of these five active molecules. Likewise, compounds **22a**, **22b**, **22d**, and **22e** showed activity close to that of novobiocin (IC_50_ = 11 μM and 27 µM, respectively) against *E. coli* and *S. aureus* topoisomerase IV, while compound **22c** (IC_50_ = 3.07 µM and 8.2 µM, respectively), containing 4-chlorophenyl substituents on the pyrrolidine ring and 5-hydroxymethyl furan moiety on the 1,2,4 oxadiazole group, was more active than the reference. Compound **22c** could be considered the lead compound for future research.


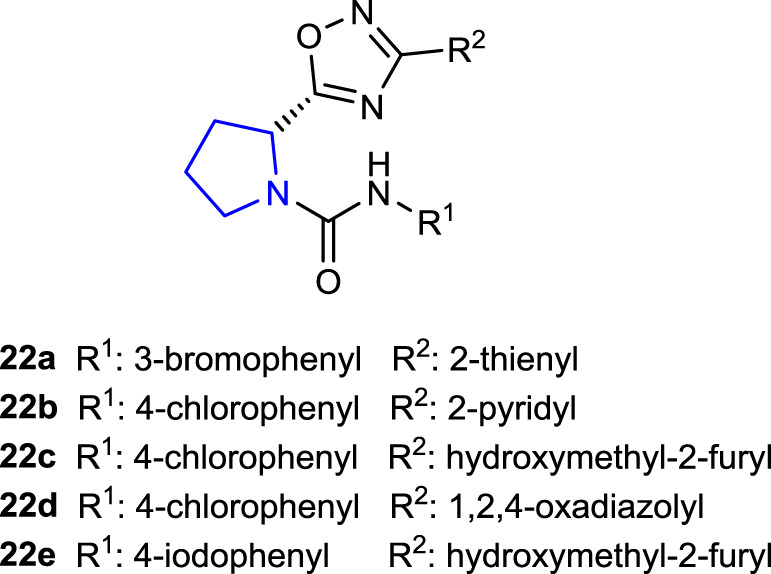


Salve and Jadhav (2021) synthesized novel pyrrolidine sulfonamide derivatives as DPP-IV inhibitors and compared their *in vitro* antidiabetic effects with those of vildagliptin as a control. Compounds **23a–d** showed 56.32%, 44.29%, 49.62%, and 66.32% inhibition, respectively. Compound **23d** with the 4-trifluorophenyl substitution on the 1,2,4-oxadiazole group exhibited the best inhibition (IC_50_: 11.32 ± 1.59 μM) against the DPP-IV enzyme among these compounds.


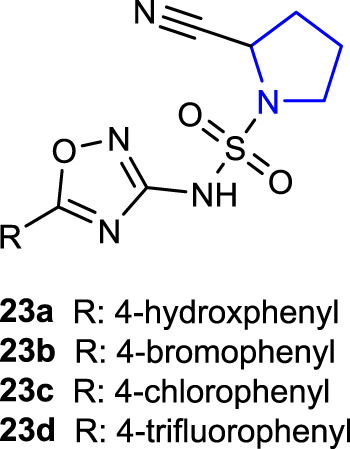


Sirin et al. (2021) synthesized the pyrrolidine-substituted 3-amido-9-ethylcarbazole derivative and performed an activity screening. Compound **24** showed an antiproliferative effect against HT-29 and SH-SY5Y cells, acetylcholinesterase inhibition activity, and antioxidant activity.


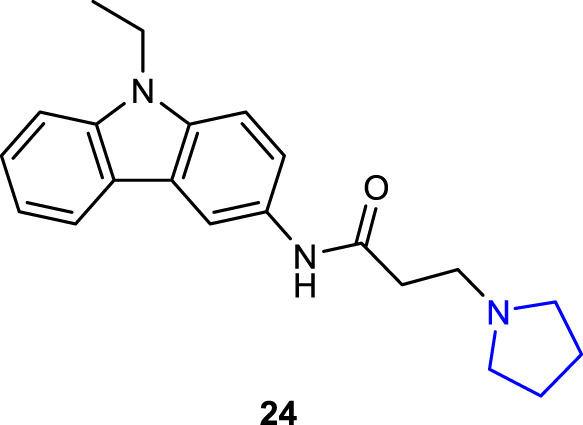


Ruan et al. (2020) designed and synthesized pyrrolidine oxadiazoles, which could be used as new anthelmintic drug candidates due to their resistance to over-used anthelmintic drugs against parasitic roundworms and reported their activity against the worm *Haemonchus contortus*. Compounds **25a** (2-CH_3_) and **25b** (3-OCH_3_) were discovered to be promising candidates against xL3 motility (IC_50_ = 0.78 µM for each), whereas compound **25c** (4-I) was identified as an efficient inhibitor of L4 development (IC_50_ = 3.2 µM). Moreover, these compounds exhibited good selectivity against mammalian epithelial cells with limited inhibition of cell proliferation at 50 mM.


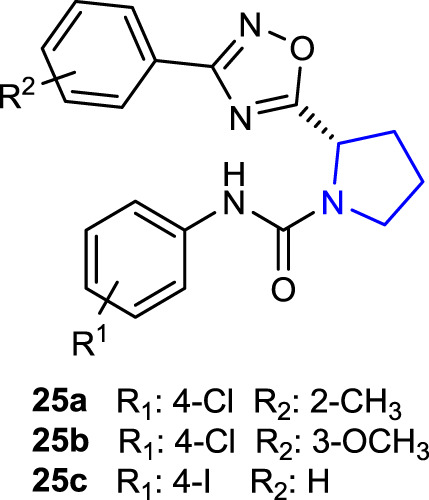


Martinez-Bailen et al. (2020) synthesized multimeric pyrrolidine iminosugars as inhibitors of important therapeutic enzymes, human β-glucocerebrosidase (GCase) and α-galactosidase A (α-Gal A), and tested them as a multivalent enzyme activity enhancer for Fabry disease. One of the nonavalent compounds showed 375-fold higher inhibition (0.20 µM) of the α-Gal A enzyme than the monovalent reference. In R301G fibroblasts from Fabry disease patients, the compound that increases the activity of the misfolded enzyme by 5.2-fold at 2.5 µM could be a candidate for a polyvalent α-Gal A activity enhancer.

Li et al. (2020) designed and synthesized pyrrolidine-containing derivatives as antagonists of the chemokine receptor CXCR4, which is responsible for essential activities in HIV infection, inflammation/autoimmune disorders, and cancer metastasis, and investigated their *in vivo* anticancer metastatic potential. Compound **26**, which contains pyridine, piperazine, and pyrimidine, together with the pyrrolidine ring, was identified as a CXCR4 antagonist with strong binding affinity to the CXCR4 receptor (IC_50_ = 79 nM) and a capability to inhibit CXCL12-induced cytosolic calcium flux (IC_50_ = 0.25 nM). In addition, to its favorable *in vitro* safety profile, compound **7** also demonstrated significantly enhanced metabolic stability in human and rat liver microsomes, as well as remarkable efficacy in a mouse cancer metastasis model. According to the experimental findings, compound **7** appeared to be promising in the development of CXCR4 antagonists.


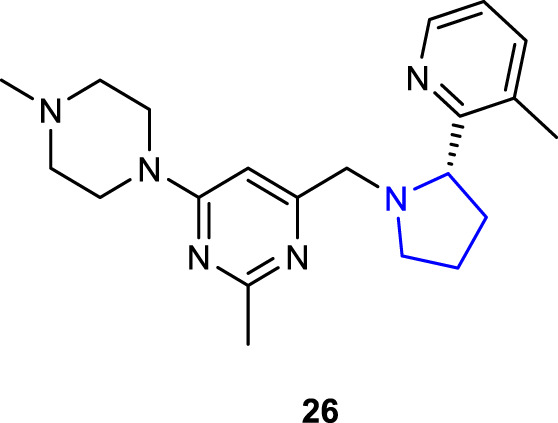


Sreekanth and Jha (2020) synthesized 1-acetyl-2-benzylpyrrolidine-2-carboxylic acid and its derivatives and evaluated their antimicrobial activities. Antibacterial activity studies were performed against some Gram-negative (*E. coli* and *P. aeruginosa*) and Gram-positive (*S. aureus* and *B. subtilis*) bacterial strains and compared with the reference antibiotic chloramphenicol. Compound **27a**, containing a butyl substituent, had the same effect (MIC: 16 μg/mL) against the Gram-positive bacteria *S. aureus* and *B. subtilis* using chloramphenicol as the reference, whereas compound **27b**, containing a propyl substituent, had the same effect (MIC: 16 μg/mL) against *S. aureus* bacterial strains using chloramphenicol as the reference. Antifungal activity studies were conducted against the *Candida albicans* strain using ketoconazole as a reference. Compound **27a** (MIC: 32 μg/mL), which is also the most active compound against the *C. albicans* strain, could be considered a promising molecule for further studies.


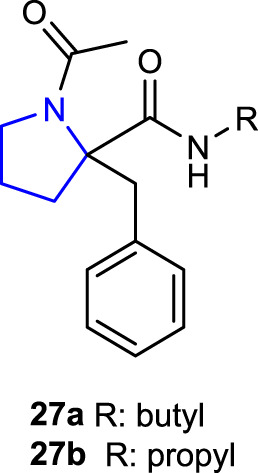


The pyrrolidine derivatives **15c–f**, bearing an indole moiety at the R^2^ position and several aromatic substituents at the R^3^ position in their structure, and compound **17**, obtained by ring arrangement exhibited twice the activity (MIC: 62.5 μg/mL) of the reference ampicillin against the *A. baumannii* strain (Belveren et al., 2019).

Since tetrazole derivatives are already known to have powerful antifungal properties, Lukowska-Chojnacka et al. (2019) synthesized tetrazoles bearing a pyrrolidine ring and tested them as prospective antifungal agents against *C. albicans*. While compound **28b** (-CH_3_ substituted) was determined to be the most effective antifungal agent, randomly selected compounds **28a**, **28d (-**Cl, -F**)**, and **28e** (-Cl, -Cl**)** exhibited little to no toxicity against the Vero cell line and *Galleria mellonella*. In the invertebrate model of disseminated candidiasis, compounds **28b** and **28c** (-F) showed the highest effectiveness against biofilm formation *in vitro* and also exhibited activity *in vivo*. Mitochondrial damage (XTT assay) and decreased adherence to the TR-146 cell line at 46.05 µM demonstrated necrotic cell death *via* fungal membrane interaction of compound **28b**.


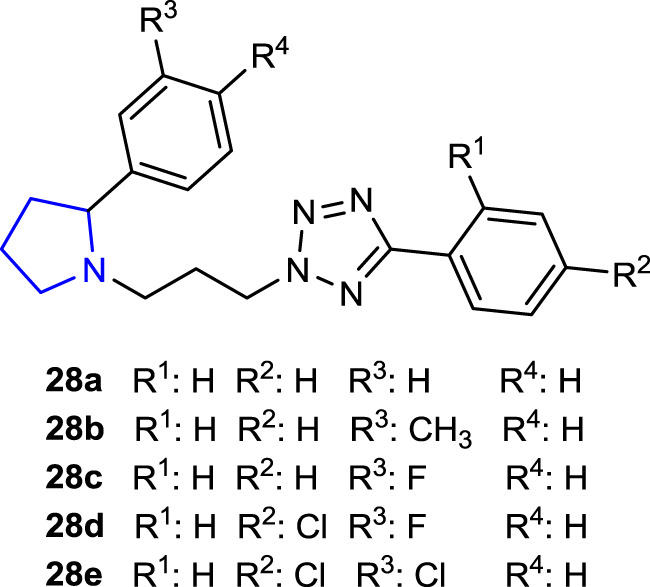


Guazzelli et al. (2019) synthesized polyhydroxylated pyrrolidine derivatives containing D-gluco- and D-galacto- units, which have the potential to inhibit glycosidase and aldose reductase enzymes and play an important role in the treatment of diabetes, a multifactorial disorder, and assessed their inhibitory properties. Among the synthesized pyrrolidine derivatives, compound **29** (57% inhibition) showed the best inhibitory property against ALR2 on an *in vitro* model of diabetic retinopathy. Furthermore, compound **29** decreased cell death processes in the photoreceptor-like 661w hyperglycemic cell line and restored physiological levels of oxidative stress, decreasing ALR2-associated ROS production.


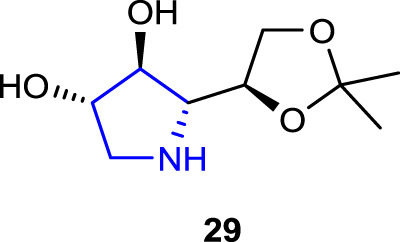


Zhou et al. (2019) synthesized a series of pyrrolidine hybrid molecules by changing the substituents at the terminal phenyl groups of pyrrolidine amide derivatives and tested their inhibitory properties against *N*-acylethanolamine acid amidase (NAAA), one of the key enzymes involved in the degradation of fatty acid ethanolamides (FAEs), specifically palmitoylethanolamide (PEA). Compounds **30a** (4-cyclohexylphenyl; IC_50_: 0.5 ± 0.1 μM), **30b** (4-(cyclohexyloxy)phenyl); IC_50_: 0.7 ± 0.2 μM), **30c** (4-phenylcyclohexyl; IC_50_: 0.48 ± 0.11 μM), and **31** (4-phenylphenyl; IC_50_: 1.5 ± 0.22 μM) displayed potent inhibitory activity toward NAAA but had no such effect toward FAAH. Compound **31** showed good selectivity (IC_50_ for NAAA: 1.5 ± 0.22 μM and for FAAH: 550 ± 70 μM) with the strongest inhibition by reversible and competitive mechanisms against the NAAA enzyme as well as high *in vitro* anti-inflammatory activity in the lipopolysaccharide (LPS)-induced acute lung injury (ALI) model.


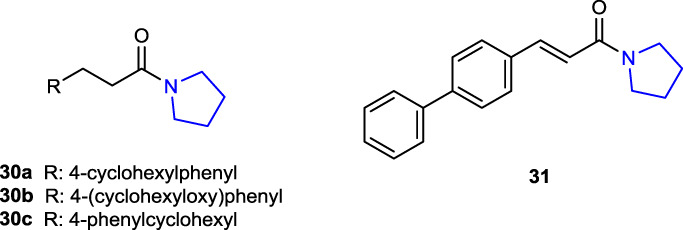


Zhang et al. (2018) designed and synthesized a series of benzofuroxane pyrrolidine hydroxamates as matrix metalloproteinase (MMP) inhibitors with nitric oxide-releasing activity and evaluated their antiproliferative activity. Among the target compounds, compounds **32a** (IC_50_ = 102 ± 31.4 and 162 ± 10.5 nM) and **32b** (IC_50_ = 182 ± 25.2 and 242 ± 29.2 nM) with 4-phenoxyphenylsulfonyl groups in their structures inhibited MMP-2 and MMP-9 enzymes better than control LY52 (IC_50_ = 266 ± 19.2 and 360 ± 30.1 nM). Meta-substituted benzofuroxane molecules exhibited more inhibitory activity than the *ortho*-substituted molecules. Furthermore, compounds were tested against A549, ES-2, HeLa, K562, MCF-7, and MDA-MB-231 cancer cells, and the findings were compared to those of the positive control LY52. Compound **32a**, which demonstrated the highest enzyme activity, also demonstrated an antiproliferative effect against HeLa cells, roughly 60 times greater than that of the control LY52 with an IC_50_ value of 3.82 ± 0.11 µM. All compounds, but **32a** (34.43 μM/L) in particular, released moderate quantities of NO.


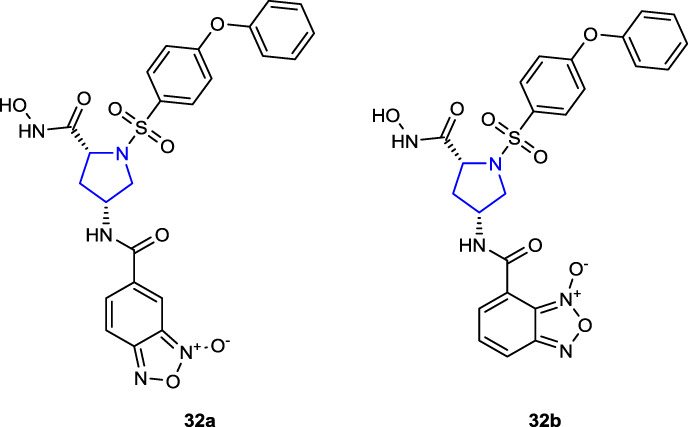


In another study, while *N*-benzoylthiourea-pyrrolidine derivatives containing *N*-phenyl **16b** and *N*-methyl **16d** groups were found to be four times more active against the *A. baumannii* strain and twice more active against *A. hydrophila* than the reference. Compound **16c**, containing the 4-chlorophenyl group, showed better antituberculosis (anti-TB) activity than the reference ethambutol. Compounds **15a** and **15b**, which are among the pyrrolidine derivatives used in the synthesis of the corresponding compounds, also showed four times higher activity (MIC: 31.25 μg/mL) against *A. baumannii* than the reference ampicillin (MIC: 125 μg/mL) (Poyraz et al., 2018).

The anti-TB activities of the thiohydantoin-pyrrolidine derivatives **35a–d** and the compounds **33a–d** and **34a–d**, used in the synthesis to access these molecules **35**, were tested against *Mycobacterium tuberculosis* using the microplate Alamar Blue assay (MABA) method. Compared to the reference isoniazid and ethambutol, the synthesized compounds did not exhibit significant anti-TB activity, while the activities of the compounds were found in the range of 62.5–125 μg/mL against the *Mycobacterium tuberculosis* H37Rv strain (Poyraz et al., 2017).

Compound **33e** (MIC: 1.95 μg/mL), which is one of the pyrrolidines synthesized for the preparation of *N*-benzoylthiourea derivatives used in the synthesis of thiazole-pyrrolidine derivatives and contains 4-chlorophenyl and indole groups in its structure, showed better anti-TB activity against *Mycobacterium tuberculosis* than the reference ethambutol (5 and 10 μg/mL). The anti-TB activity of compound **36** (MIC: 0.24 μg/mL), which was obtained by condensation of compound **33e** with benzoyl isothiocyanate, was found to be eight times more potent than that of the starting compound **34e** and better than that of the reference anti-TB drugs isoniazid and ethambutol. In addition, the anti-TB activity of compound **36**, obtained by the addition of 4-bromomethoxy acetophenone to compound **34e**, was found to be 0.48 μg/mL. Moreover, the antimicrobial activities of the compounds were tested against *S. aureus*, *E. coli*, *A. baumannii*, *B. subtilis*, and *A. hydrophila* strains. Compound **15c**, which contains an indole heterocycle, together with a fused bicyclic pyrrolidine ring, was twice more active against *A. baumannii* than the reference antibacterial agent ampicillin (MIC: 125 μg/mL) with a MIC value of 62.5 μg/mL (Belveren et al., 2017).


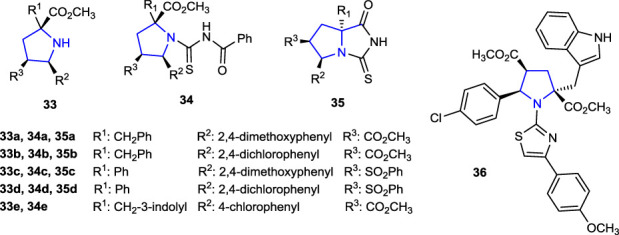


Dobrova et al. (2016) synthesized nickel(II), palladium(II), and copper(II) complexes of pyrrolidine thiosemicarbazone hybrids, evaluated their *in vitro* anticancer potential against A549 (lung cancer), CH1 (human ovarian carcinoma), and SW480 (colon cancer) cancer cell lines and noncancerous murine embryonal fibroblasts (NIH/3T3), and studied the cell death mechanism using the flow cytometry method. The outcomes were evaluated against a cisplatin control group. The thiosemicarbazone pyrrolidine–copper(II) complexes, with two pyrrolidine rings in their structure, were the most potent anticancer compounds against three cancer lines among the synthesized ligands and complexes. Copper complex **37a** (IC_50_: 0.99 ± 0.09) was approximately three times more potent than cisplatin used as a control (IC_50_: 3.5 ± 0.3) against the SW480 cancer cell line, while copper complex **37b** (IC_50_: 3.7 ± 0.1) showed approximate activity to cisplatin. The results of the research revealed that altering the coordinating groups of the ligands enhanced their antiproliferative activity.


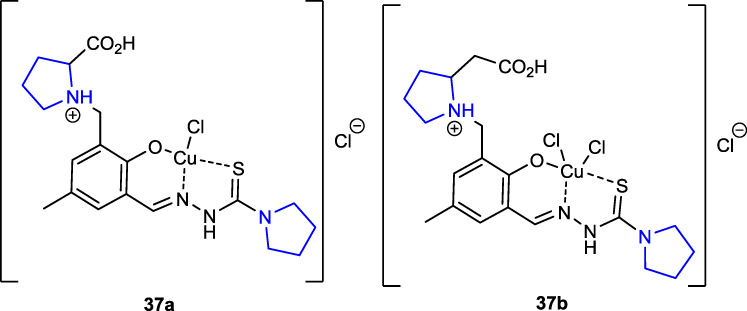


Kumar et al. (2016) synthesized sulfonylamino pyrrolidine derivatives and evaluated their *in silico* EGFR potential as well as their antibacterial and antifungal activities. Antibacterial activity studies were compared with those of reference cefaclor against *S. aureus*, *E. coli*, and *P. aeruginosa* bacteria. Compound **38**, which contains two nitrophenyls together with the pyran ring as a substituent in its structure, showed the best activity against three bacteria [*S. aureus* (MIC: 3.11 μg/mL), *E. coli* (MIC: 6.58 μg/mL), and *P. aeruginosa* (MIC: 5.82 μg/mL)]. Antifungal activity studies were also performed against *A. niger* and *C. albicans* fungal strains using ketoconazole as the reference. Compound **38** also showed the highest activity against the respective fungal strains [*A. niger* (MIC: 1.78 μg/mL) and *C. albicans* (MIC: 3.18 μg/mL)]. Compared to the reference, *in silico* studies showed that the compounds could serve as EGFR inhibitors.


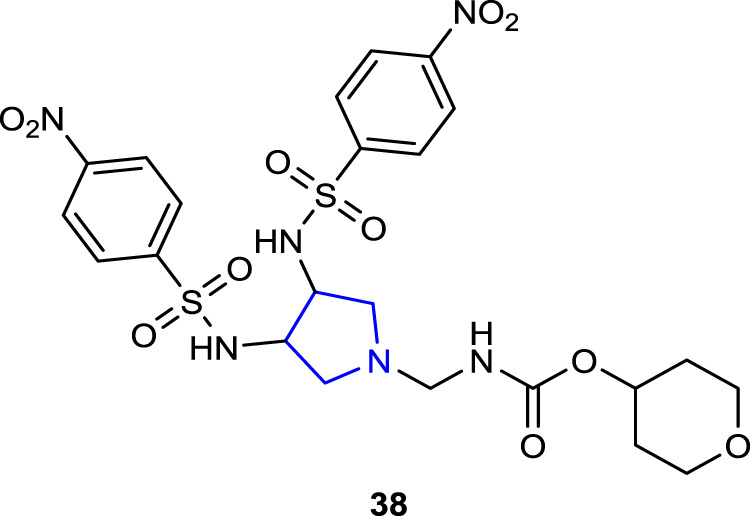


Huang et al. (2015) synthesized a series of 1-[(1*R*,2*S*)-2-fluorocyclopropyl]naphthyridone derivatives containing oxime-functionalized pyrrolidine and evaluated their antimicrobial activities. The antituberculosis (TB) activity of pyrrolidine derivatives was compared with that of the reference drugs isoniazid and rifampicin using the MABA method against the *Mycobacterium tuberculosis* (MTB) H37Rv ATCC 27294 (MTB-1) strain. Compounds **39a**, **39c,** and **39d**, which contain 4-F, 4-CH_3_, and 2′,3′-methylenoxyl substituents, respectively, were found to be the most active compounds against the MTB-1 strain with a MIC value of <0.25 μg/mL. In addition, these most active compounds against the clinically isolated MDR-MTB 6133 (MTB-2) strain resistant to isoniazid and rifampicin showed much better anti-TB activity than the references (MIC: 0.043, 0.03, and 0.054 μg/mL, respectively). The antibacterial activity of the compounds was tested against a series of Gram-negative (*E. coli*, *K. pneumoniae*, and *P. aeruginosa*) and Gram-positive (*S. aureus*, MSSA: methicillin-sensitive *S. aureus*, MRSA: methicillin-resistant *S. aureus*, MSSE: methicillin-sensitive *S. epidermidis*, MRSE: methicillin-resistant *S. epidermidis*, *S. pneumoniae*, *E. faecalis*, and *E. faecium*) strains. Ciprofloxacin (CPFX), levofloxacin (LVFX), and moxifloxacin (MXFX) were used as references. Compounds **39b**, **39c,** and **40a**–**c** showed better activity (MIC: <0.008–32 μg/mL) against Gram-positive bacteria than against Gram-negative bacteria than the references (MIC: 0.125–>128 μg/mL).


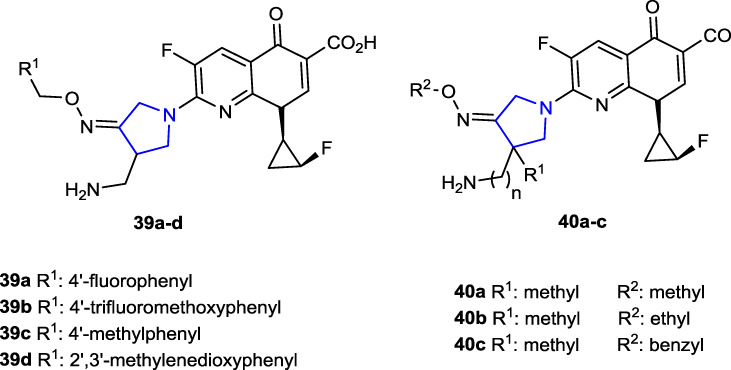


## 3 Spiropyrrolidine-based hybrids

Liu et al. (2021) synthesized spiropyrrolidine oxindole derivatives carrying nitroisoxazole as novel GPX4/MDM2 inhibitors inhibiting breast cancer cell proliferation. Among the synthesized compounds, compound 6-Cl-substituted **41** (*K*i: 0.24 ± 0.06 µM) exhibited the highest activity in suppressing MDM2-mediated p53 degradation as well as GPX4 levels in MCF-7 breast cancer cells.


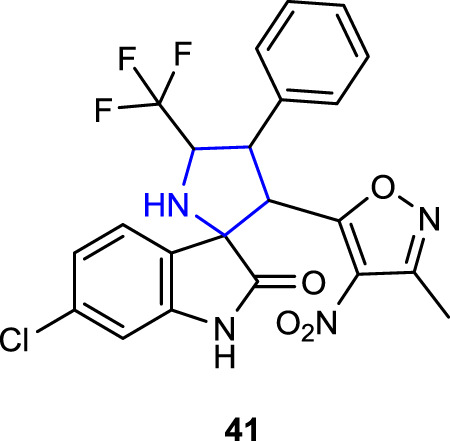


Toumi et al. (2021) designed rhodanine-substituted spirooxindole pyrrolidine compounds and tested their ability to inhibit the α-amylase enzyme, which is related to diabetes. While almost all compounds inhibit the α-amylase enzyme at a certain level, the compounds **42c** (IC_50_: 1.59 ± 0.08 μg/mL), **42d** (IC_50_: 1.67 ± 0.09 μg/mL), **42e** (IC_50_: 1.63 ± 0.09 μg/mL), and **42f** (IC_50_: 1.57 ± 0.10 μg/mL), bearing R_1_: NO_2_, H, H, and Br; R_2_: H, benzyl, benzyl, and H; and Ar: Br, F, Cl, and Cl substituents, respectively, showed approximately the same level of inhibition as the reference acarbose (IC_50_: 1.56 ± 0.09 μg/mL). Compound **42a** (IC_50_: 1.49 ± 0.10 μg/mL), with a 4-bromo substituent on the isatin ring in its structure, was more active than the standard acarbose. In subsequent investigations of *in vivo* hypoglycemic activity, **42a–c** and **42f** were the most active compounds that decreased the blood glucose levels. The findings suggested that *N*-substituted groups attached to isatin and substituents on the phenyl ring may both affect enzyme function.


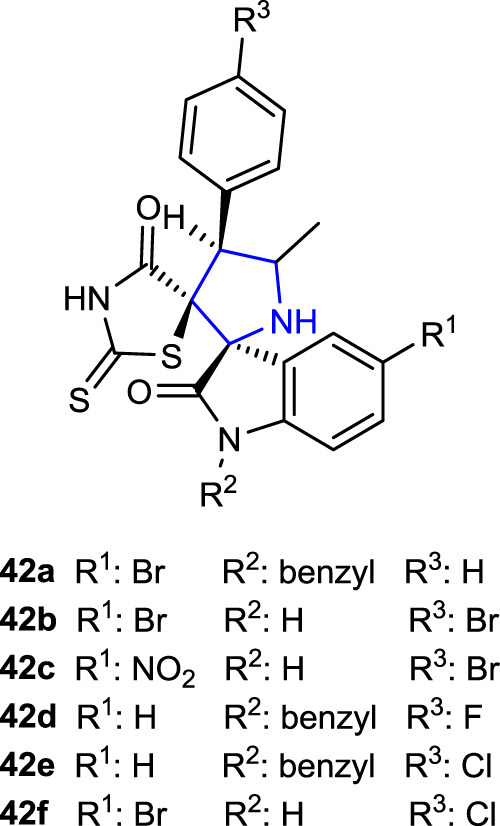


Islam et al. (2020a) synthesized two series of spiropyrrolidine-thiazolo-oxindole derivatives, namely, dibromo-substituted and non-substituted oxindole, and evaluated their anticancer activity against HepG2, MCF-7, and HCT-116 cell lines. The results were compared to those of cisplatin and reported. Thus, compounds 2,4-dichlorophenyl-substituted **43a** (IC_50_: 0.85 ± 0.20 μg/mL) and 4-bromophenyl-substituted **43b** (IC_50_: 0.80 ± 0.10 μg/mL) were discovered to be roughly 11 times more active against the HepG2 cell line than the reference (IC_50_: 9.00 ± 0.76 μg/mL) among the non-substituted oxindole compounds. In addition, compound **43a** (IC_50_: 2.00 ± 0.60 μg/mL) was more active than the reference compound against the HCT-116 cell line, whereas compound **43b** (IC_50_: 3.00 ± 0.50 μg/mL) displayed the same activity. One of the dibromo-substituted oxindole derivative compounds, 3-NO_2_-substituted **43c** (IC_50_ HepG2: 5.00 ± 0.66, IC_50_ MCF-7: 4.00 ± 0.29, and IC_50_ HCT-116: 2.80 ± 0.20 μg/mL), showed higher activity against all three cell lines than the reference, as well as approximately twice better activity than cisplatin (IC_50_: 9.00 ± 0.29 μg/mL) against the MCF-7 cell line.


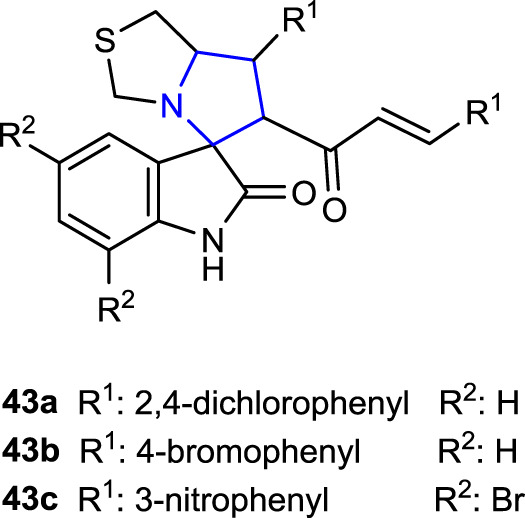


Bolous et al. (2019) designed and synthesized spirooxindole pyrrolidine-linked indole and imidazole heterocyclic hybrids and evaluated the advanced antifungal activity against clinically isolated fungal strains for compound **44**, which was found to be the most active antifungal agent after broad-spectrum antifungal screening. In the tests, compound **44** containing OCF_3_ and 2-Cl was found to be the most active compound against the *C. albicans* strain with a MIC value of 4 μg/mL. Compound **44** was then tested against pathogenic strains of *C. albicans*, *C. glabrata*, *C. tropicalis*, *C. parapsilosis*, and *Cryptococcus neoformans* isolated from humans. These fluconazole-resistant organisms were inhibited by compound **44**. In addition, compound **44** reduced the activity of the *C. albicans* strain, which gained resistance to antifungal agents by forming filaments and biofilms, by up to 25% at concentrations of 32 μg/mL and 64 μg/mL. The MTS assay was also used to determine the non-toxicity of compound **44** to the HCT116 mammalian cell line *in vitro*.


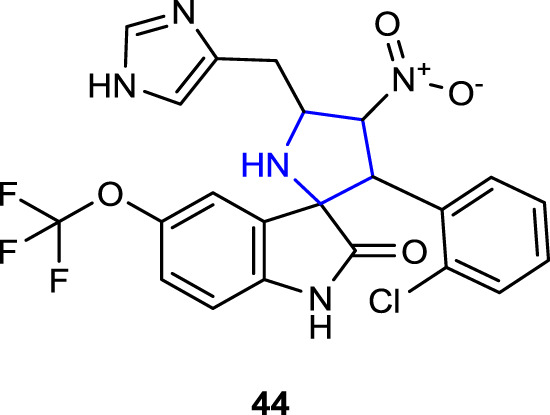


Huang et al. (2018) synthesized a series of spirooxindole pyrrolidine derivatives and evaluated their antibacterial activities against *E. coli* and *S. aureus* and inhibitory potency against the AChE enzyme. The most active compounds against *E. coli* bacteria were compounds **45b** (R_1_: Cl and R_2_: CH_2_Ph) and **27c** (R_1_: CH_3_, R_2_: H, and Ar: 4-CH_3_C_6_H_4_). The compounds did not show significant activity against the bacterial strains. Compounds **45a** (R_1_: Cl and R_2_: CH_2_Ph; 94.66% ± 3.26%) and **45d** (R_1_: Cl, R_2_: butyl, and Ar: 4-CH_3_C_6_H_4_; 64.95% ± 1.64%) showed the strongest inhibition against the AChE enzyme compared to the reference tacrine. The IC_50_ values of compounds **45a** and **45d** were found to be 69.07 ± 10.99 µM and 111.38 ± 10.11 µM, respectively.


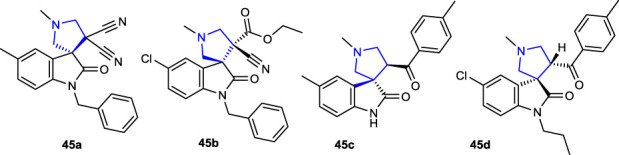


Kumar et al. (2018) synthesized a series of spiropyrrolidine analogs in a stereoselective manner and evaluated their anticancer effects against cancer and non-cancer cell lines. In anticancer activity studies, the mechanism of action of the compounds was investigated using non-cancer (PCS-130-010 and BRL-3A) and cancer (A549 and Jurkat cell types) cell lines and compared with the reference anticancer drug camptothecin (CPT) for 24- and 48-h periods. The compounds showed no significant loss of cell viability and were not toxic to non-cancer cells. Compound **46a** with a 4-Br substitution was the most active, whereas compound **46b** with a 4-OCH_3_ substituent was the least active. Moreover, cell death was noted to be an apoptotic pathway mediated by the increased activation of caspase-3 proteins, while halogen-substituted pyrrolidines were found to be more active than the others.


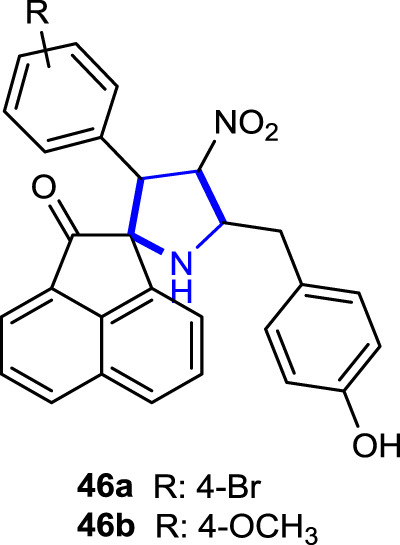


Millington et al. (2018) synthesized spirotryprostatin analogs **47a–g** and **48a, b**, known for their anticancer properties and containing a spirooxindole structure, by metal-catalyzed imine azomethine ylide cycloaddition reactions following by the Heck reaction.


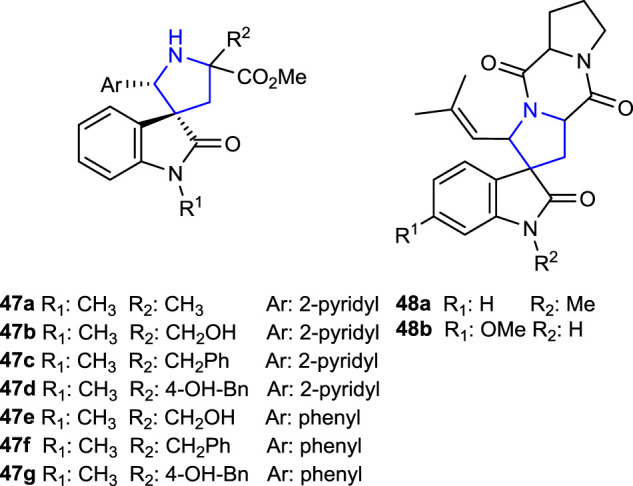


Bharkavi et al. (2016) developed dispiro indeno pyrrolidine derivatives and tested their AChE inhibition, antituberculosis, and anticancer activity. Compound **49c** (IC_50_: 1.07 μg/mL), which contains a 2,4-dichlorophenyl group, was found to be approximately 12 times more active than the reference cycloserine (IC_50_: 12.47 μg/mL) and 36 times more active than the reference pyrimethamine (IC_50_: 37.35 μg/mL) against *Mycobacterium tuberculosis* H37Rv. In addition, compound **49a** (IC_50_: 55.79 ± 2.60 μg/mL), containing a 4-fluorophenyl substituent, exhibited the same activity as the standard doxorubicin (IC_50_: 55.91 ± 1.34 μg/mL) against CCRF-CM cell lines but did not show significant activity against HT29 and MCF7 cell lines. Moreover, compound **49b** (IC_50_: 1.16 ± 0.1 μmol/L), containing 4-methoxyphenyl, was the most active compound against the AChE enzyme compared to the reference donepezil.


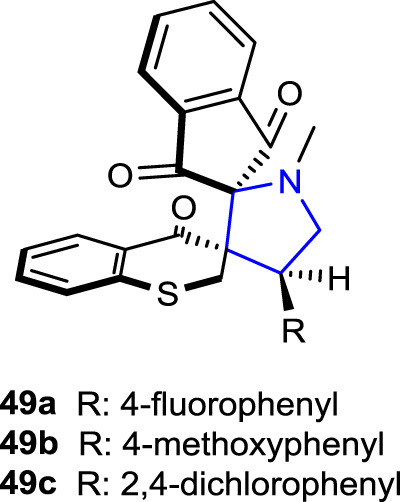


## 4 Pyrrolidinedione derivatives

Al-Zharani et al. (2022) synthesized pyrrolidine-2,4-diones and evaluated mosquito larvicidal activities against *Culex quinquefasciatus* and compared with the reference permethrin. Compounds **50a** (LD_50_ = 26.06 μg/mL), containing acetamide in its structure, and **50b** (LD_50_: 26.89 μg/mL), containing a benzamide moiety, showed larvicidal activity comparable to the reference (LD_50_ = 26.14 μg/mL). The experimental results and the highest docking scores for compounds **50a** and **50b** against the 3OGN protein are consistent.


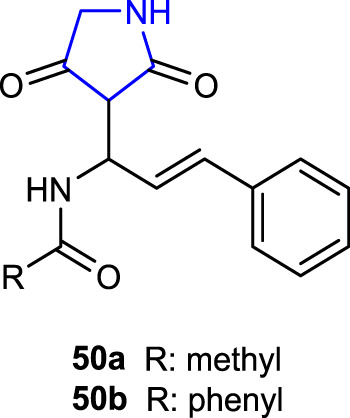


Kocabaş et al. (2021) synthesized a series of pyrrolidine-thiazole derivatives and evaluated their antibacterial activities and cytotoxicity**.** The antibacterial activities of the compounds were tested using the microdilution method against *E. coli*, *S. typhimurium*, *B. cereus*, and *S. aureus* strains, and their cytotoxicity was tested on L929 cells using the XTT method. Compound **51a** (MIC: 21.70 ± 0.36 and 30.53 ± 0.42 μg/mL), containing the 4-fluorophenyl substituent attached to the thiazole ring, was compared with the reference gentamicin (MIC: 22.65 ± 0.21 and 22.17 ± 0.47 μg/mL), showing the best activity against *B*. *cereus* and *S. aureus*. In addition, compounds **51a**, **b**, containing 4-bromophenyl and 4-fluorophenyl substituents, respectively, did not show toxicity at the concentrations performed.


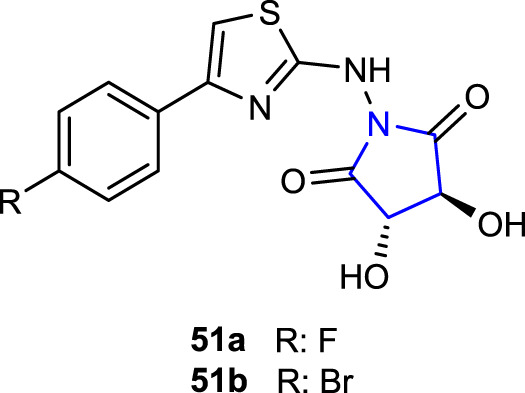


Chen et al. (2021) incorporated natural herbicide tetramic acids bearing pyrrolidine-2,4-dione with ether structures containing alkoxyalkyl and phenoxyethyl groups, which are widely known for their herbicide, fungicidal, and insecticide effects, into a single molecule and immediately evaluated their herbicidal activities using the Petri dish culture method in barnyard grass (*Echinochloa crus-galli*) and rape (*Brassica campestris*) model plants. Compounds **52a** (84%), with a 4-CH_3_ substituent, and **52b** (65.6%), with 2-OCH_3_, showed the highest inhibition rates.


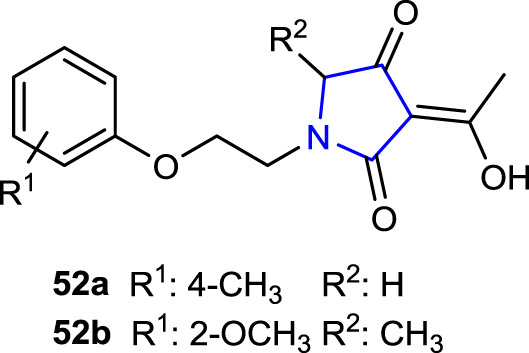


Góra et al. (2020) synthesized a series of 3-(3-methylthiophen-2-yl)-pyrrolidine-2,5-dione hybrids and evaluated their anticonvulsant and antinociceptive activities *in vivo* in animal models using MES, *sc*PTZ, and psychomotor tests (6 Hz). The effects of compounds **53**, which include 2-morpholinoethyl, 3-morpholinopropyl, 3-CF_3_Ph, and 3,4-dichlorophenyl substituents in their structures, on GABA transporters, sodium, and calcium channels were studied to determine the anticonvulsant action mechanisms. Compound **53b** was the most active compound, with an ED_50_ of 62.14 mg/kg in the MES test and 75.59 mg/kg in the 6 Hz test compared to the reference valproic acid and ethosuximide. In addition, the hot plate and writhing tests evaluated the antinociceptive activities of these active compounds. The results showed that compound **53d** demonstrated an analgesic effect comparable to that of aspirin at a dose of 30 mg/kg in the writhing test, whereas compounds **53c** and **53d** demonstrated central analgesic activity at the same dose in the hot plate test.


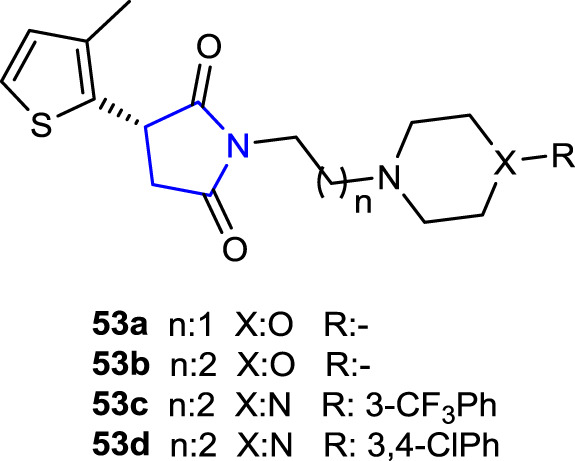


Gerokonstantis et al. (2020) synthesized 2-pyrrolidinone and pyrrolidine derivatives and evaluated the enzyme inhibition activities of autotaxin (ATX) isolated from melanoma cells, which are responsible for various pathological inflammations such as fibrosis, cancer, liver toxicity, and thrombosis due to hydrolysis of lysophosphatidylcholine (LPC). Among the compounds, compounds **54a** (IC_50_: 0.05 µΜ), **54b** (IC_50_: 0.12 µΜ), and **54c** (IC_50_: 0.18 µΜ) (of which **54a**, **b**, **d** had *S* configuration and **54c** had *R* configurations), bearing benzyl, 4-fluorobenzyl, and benzyl, respectively, and **54d** (IC_50_: 0.035 µΜ), which are boronic acid derivatives, could be considered the most potent ATX inhibitors. Compound **54d** (IC_50_: 0.035 µΜ) with *S* configuration was the most active among these compounds.


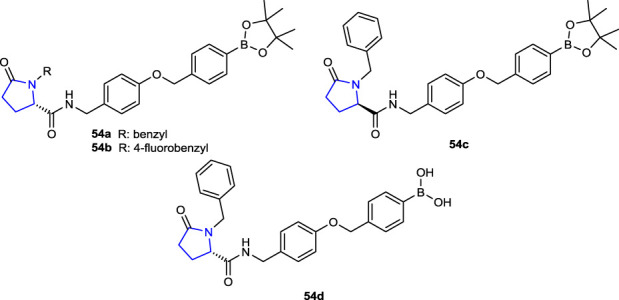


## 5 Natural sources containing the pyrrolidine ring

**FIGURE 3:**
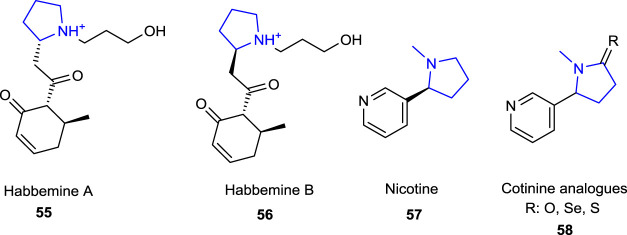
Naturally occurring pyrrolidine alkaloids.

Pyrrolidine alkaloids refer to five-membered nitrogen-containing heterocyclic ring systems and exhibit a wide variety of biological and pharmacological activities. Because of the versatile biological importance of pyrrolidine alkaloids, there are many reports and excellent reviews dealing with the source of these alkaloids, together with the chemistry and biological importance of pyrrolidine-type alkaloids **55**–**58** (Figure 3) (O'Hagan, 2000; Ibrahim SRM, 2015; Hosseinzadeh et al., 2018; Islam and Mubarak, 2020b; Jeelan Basha et al., 2022; Bhat et al., 2023b). Some of the pyrrolidine alkaloids known for their neurological and antioxidant effects (e.g., habbemines A and B and nicotine), together with a potential protective agent for newly prepared nicotine derivatives, were reported by Malczewska-Jaskola et al. (2016) against *in vitro* oxidative hemolysis and morphological injury of human erythrocytes.

A marine-originated pyrrolidine alkaloid, scalusamide A **59**, reported by Tsuda et al. (2005), which was obtained from the gastrointestinal tract of a marine fish, isolated from the cultured broth of the fungus *Penicillium citrinum*, and characterized by analytical techniques, was screened for its antifungal and antibacterial activities, which showed potential inhibition when tested against *Penicillium brevicompactum*, *Cryptococcus neoformans*, and *Micrococcus luteus*.


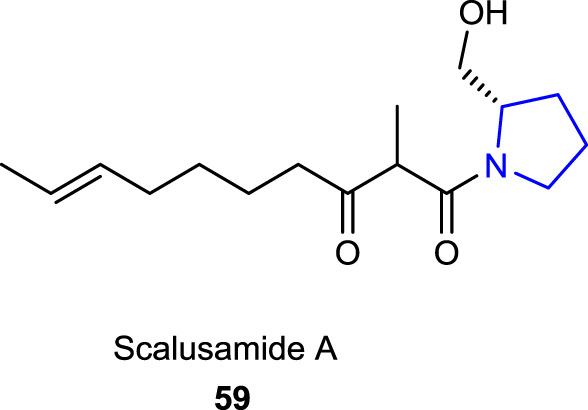


Some spiro-based structural skeleton pyrrolidine alkaloids and amathaspiramide analogs A-F **60** were isolated from marine bryozoan *Amathia wilsoni* by Morris and Prinsep (1999), and Shimokawa et al. (2016) studiedtheir antiproliferative activity against HCT116, Pc-3, MV4-11, and MiaPaCa-2 cell lines.


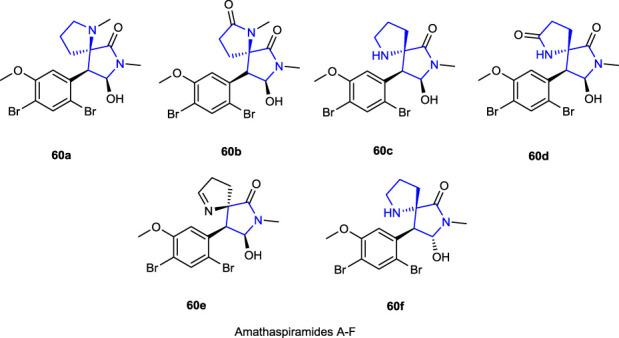


Shi et al. (2016) reported some biologically active new pyrrolidine alkaloids, ficushispimines A **61a** and B **61b**, which were isolated from the twigs of *Ficus hispida*, exhibiting glucosidase inhibitory effects.


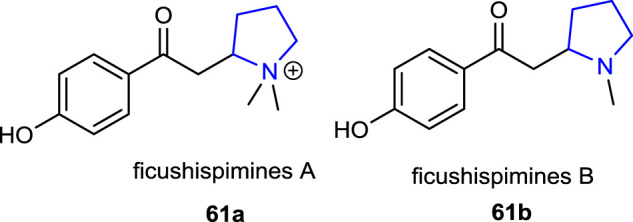


One of the pharmacologically and biologically important pyrrolidine alkaloids is the drug named anisomycin **6** (also known as flagecidin). It is an antibiotic isolated from various *Streptomyces* species (e.g., produced by bacteria *Streptomyces griseus* and *Streptomyces hygrospinosus*). This naturally occurring monohydroxypyrrolidine alkaloid acts to inhibit bacterial protein synthesis. It is used as an antiparasitic, antimicrobial, antineoplastic, and antifungal agent and as a DNA and protein synthesis inhibitor (blocks peptide bond formation). Anisomycin **6** has also been used for the treatment of trichomoniasis and amoebic dysentery and has shown *in vitro* potential antitumor activity against human tumor cell lines (Zheng et al., 2017).

Other biologically and pharmaceutically important tropane alkaloids, belonging to the pyrroline classes of plant alkaloids (subgroup of pyrrolidine), result from metabolic diversity and occur in phylogenetically distinct plant families that possess the tropane core structure [e.g., tropinone **62**, scopolamine **63**, and cocaine **64**] (Kim et al., 2016). The anticholinergic drug scopolamine, a tropane alkaloid that could be obtained naturally or synthetically, was previously prescribed to treat motion sickness and postoperative nausea and vomiting (Apfel et al., 2010). Tropinone alkaloid, synthesized as a synthetic precursor of atropine, has the same tropane core structure as cocaine and atropine (Drager, 2006).


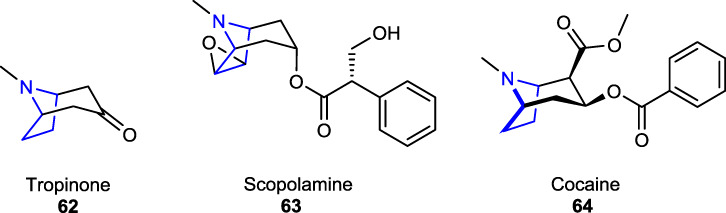


2-Alkylpyrrolidine derivatives [(*R*)-bgugaine **65**, (*R*)-irnidine **66**, and (*R*)-irnidine **67**] are versatile pyrrolidine alkaloids isolated from the tubers of *Arisarum vulgare*, which revealed significant bioactivities such as apoptosis, cytolysis effects, and antimycotic, antimitotic, antibacterial, and antifungal activities (Melhaoui et al., 1992; Melhaoui et al., 1993; Melhaoui and Belouali, 1998; Rakba et al., 1999; Lamkadem et al., 2004; Majik et al., 2008), and cytotoxic effects and showed hepatotoxin activity in rat and human liver cell culture (Rakba et al., 1999). In addition, some of the (*R*)-enantiomers of these alkylpyrrolidine alkaloids have been reported to possess a binding affinity for DNA, together with induced significant DNA damage in a human hepatoblastoma (HepG2) cell line (Melhaoui, 1998; Rakba et al., 1999), and revealed electrophysiologic activity against MRC-5 fibroblasts at 10 µM (2.81 μg/mL) (Lamkadem et al., 2001).


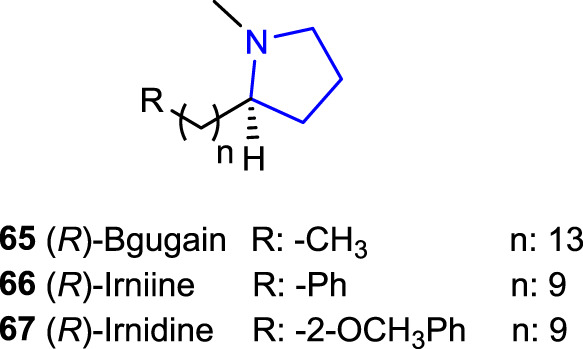


Some naturally occurring pyrrolidine dicarboxylate derivatives, known as kainoids, exhibit both excitatory and excitotoxic activity and are used as pharmacological probes. Kainic acid/kainite **68**, one of the kainoids which was first isolated from the seaweed *Digenea simplex* (Chekan et al., 2019), is a potent neuroexcitatory amino acid agonist that acts by activating receptors for glutamate as a neurotransmitter in the central nervous system (Carlson, 2013).


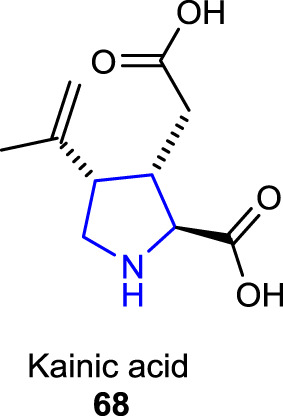


A naturally occurring neurotoxin, domoic acid **69**, an analog of kainic acid-type pyrrolidine, which is produced by the alga called *Pseudo-nitzschia australis*, affects the brain and causes seizures and memory impairment and acts as a potent activator of the kainic receptors in the CNS (Clayton et al., 1999; Carcache et al., 2003). Some congeners of domoic acid (isodomoic acids) **70**–**73** were isolated from a red alga called *Chondria armata*, and their structures were deduced to be (*E*,*E*)- and (*Z*,*E*)-isomers of 2-carboxy-4-(5-carboxy-l-methyl-2-hexenylidene)-3-pyrrolidineacetic acid **74** after their structural determination (Zaman et al., 1997).


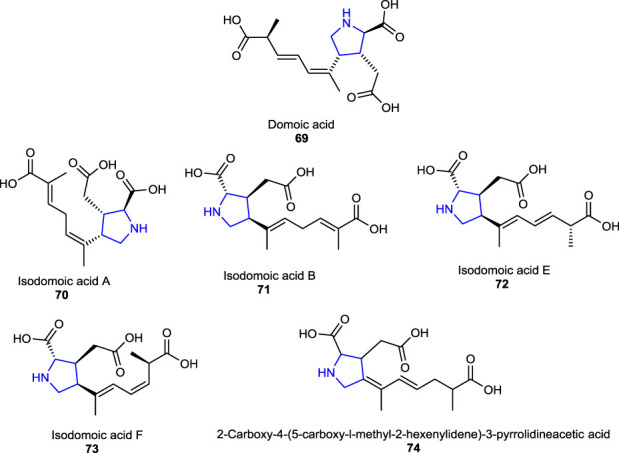


Some of the other pyrrolidine dicarboxylate derivatives, known as acromelic acids (A-C) **75**–**77**, bearing a pyridone moiety, were isolated from a poisonous mushroom *Clitocybe amoenolens/Clitocybe acromelalga* and act as a neurotoxin, together with a role as an NMDA (*N*-methyl-D-aspartic acid) receptor agonist. Acromelic acid (C) was found to exhibit a lethal toxic effect on mice at a dose of 10 mg/kg (Fushiya et al., 1990; Carcache et al., 2003)**.**


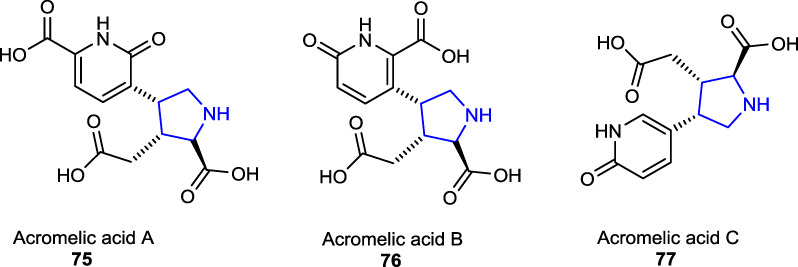


Recently, two tetracyclic functionalized pyrrolidine-type alkaloids, senecipyrrolidine **78** and emiline **79**, were isolated from *Jacobaea gigantea* (*Jacobaea*/*Senecio* genera) (Asteraceae) by Mezache et al. (2019) and characterized using analytical techniques. The herbal plant *J. gigantea* is also known as an endemic herb that was found to grow in Algeria, Morocco, and Tunisia.


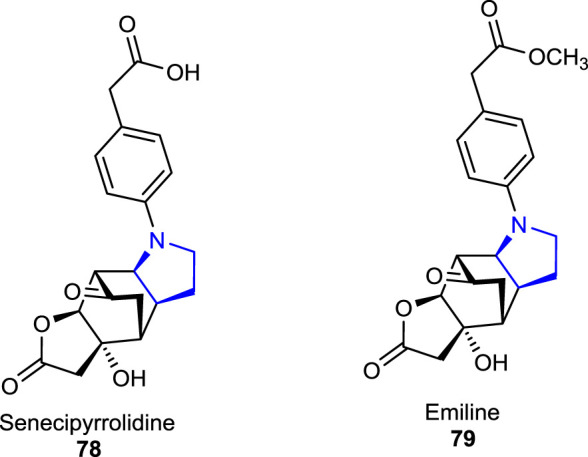


Three other naturally occurring pyrrolidine alkaloids, namely, acanthophoraine A **80**, acanthophoraine B **81**, and acanthophoraine C **82**, were isolated from a red alga called *Acanthophora spicifera*, and their antibacterial activities were screened against the bacterial strains, including *E. faecalis*, *S. aureus*, *P. aeruginosa*, *K. pneumoniae*, *A. baumannii*, and *E. coli*, and no desirable effects were found on the tested bacteria (Lin et al., 2020; Guan et al., 2021).


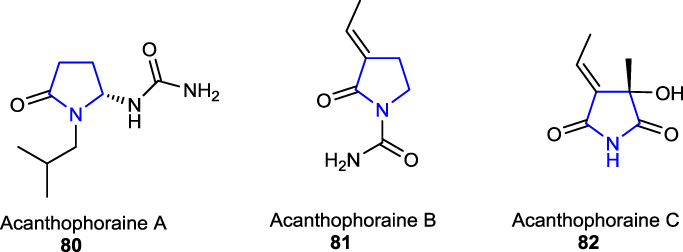


The hygrolines **83** and **84** and pseudohygrolines **85** and **86** are 2-substituted pyrrolidine alkaloids with two chiral centers constituting a 1,3-aminoalcohol unit, isolated from Solanaceae *(S. tricolor)*, *Schizanthus hookeri*, *Carallia brachiate*, and genera *Schizanthus* and *Erythroxyloncoca*. These structurally simple 2-substituted naturally occurring pyrrolidine alkaloids are important pharmacophores due to their intriguing biological and pharmacological activity. Some of its derivatives have been found to show significant hallucinogenic characteristics and displayed antitrypanosomatid and antiplasmodial activities (Cretton et al., 2017; Christen et al., 2020; Cretton et al., 2021).


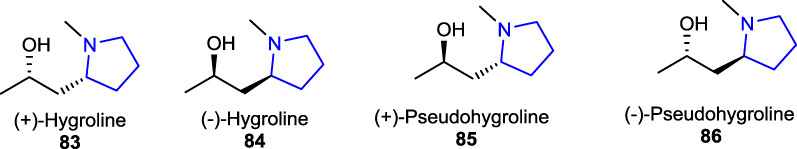


Ibrahim SRM (2015) reported potentially bioactive aegyptolidine A **87** and B **88** pyrrolidine alkaloids which were isolated from *Aspergillus aegyptiacus* (a species of the fungus in the genus *Aspergillus*). The studies have shown that these two alkaloids exhibited weak cytotoxic activity against the murine lymphoma cancer cell (L5178Y) line.


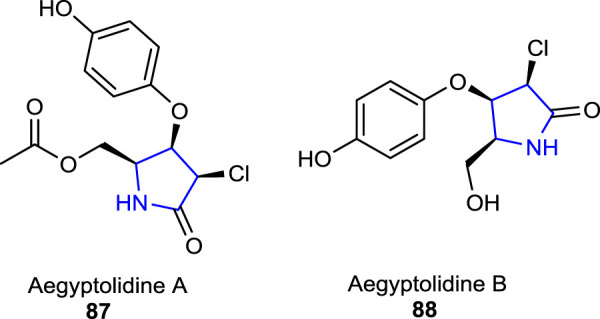


There are many structurally identified polyhydroxy pyrrolidine alkaloids that possess glycosidase inhibitory properties, which have been isolated from natural sources (Watson et al., 1997; Shibano et al., 1998; Elbein and Molyneux, 1999; Shibano et al., 1999; O'Hagan, 2000). Some of the well-known extensively studied representative examples of these alkaloids are broussonetines **89**–**96** (*Broussonetia* species), mainly isolated from the branches of *Broussonetia kazinoki* Sieb. (*Moraceae*). Shibano et al. reported the isolation, structural determination, and bioactivities of these series of pyrrolidine alkaloids called broussonetines A–H **89**–**96** and screened them for their inhibitory activities on some glycosidases (Shibano et al., 1997; Shibano et al., 1998).


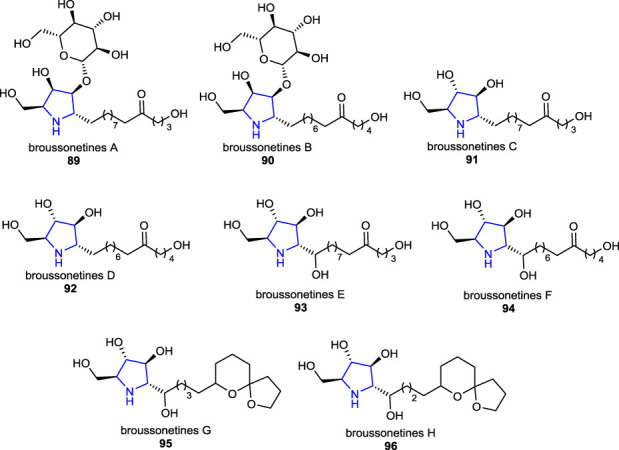


## Conclusion

This review focuses on the current progress in pyrrolidine derivatives, which show multiple pharmacological activities and functions in the treatment of several health disorders. Pyrrolidine-based therapeutic candidates are currently used in the therapy of some diseases. In view of the aforementioned information, combining pyrrolidine compounds, which are known to exhibit many biological activities, with different bioactive heterocyclic structures *via* molecular hybridization will contribute to the development of alternative molecules to drugs that are used in the treatment of diseases and have various limitations. The pyrrolidine skeleton continues to provide new formulas of synthetic and natural compounds with diverse biological activities. Many SAR studies have confirmed the crucial role of this heterocycle in the effective interaction with the natural target. It is noteworthy that, despite the overwhelming variety of substituents anchored to it, a selected number of them are ready to promote beneficial biological interaction. It is important not to discard minor effects in biomedical applications because the fine tuning of the atomic arrangement of the radicals bonded to the ring can raise a very important interaction. The chiral natural pool also provides new compounds, which can serve as starting points to design potent inhibitor agents and can also be useful for the inspiration for the search of many other drug surrogates. This field has wide scope, and extensive work is necessary to develop new drugs.

## Figures and Tables

**Table:** 

**ATCC**	American Type Culture Collection
**ALR2**	Aldose reductase
**A549**	Lung cancer cell line
**ALI**	Acute lung injury
**AChE**	Acetylcholinesterase
**ATX**	Autotaxin
**BuChE**	Butyrylcholinesterase
**CF**	Cystic Fibrosis
**CNS**	Central nervous system
**CPT**	Camptothecin
**CH1**	Human ovarian carcinoma
**CXCR4**	Chemokine receptor
**CXCL12**	Stromal cell-derived factor-1 or SDF-1
**DPP-IV**	Dipeptidyl peptidase-4
**EGFR**	Epidermal growth factor receptor
**ES-2**	Ovarian clear cell carcinoma cell
**FGFR-4**	Fibroblast growth factor receptor
**FDA**	Food and Drug Administration
**FAEs**	Fatty acid ethanolamides
**FAAH**	Fatty acid amide hydrolase
**GABA**	Gamma aminobutyric acid
**GCase**	β-Glucocerebrosidase
**HT-29**	Human colorectal adenocarcinoma cell line
**HIV**	Human immunodeficiency virus
**HeLa**	Human cervical carcinoma cell
**HCT-116**	Colon cancer cell line
**HT29**	Caucasian colon adenocarcinoma grade II
**HepG2**	Liver cancer cell line
**JAK2**	Janus family of kinases
**K562**	Chronic myelogenous leukemia cell
**L5178Y**	Murine lymphoma cancer cell
**LPC**	Lysophosphatidylcholine
**LPS**	Lipopolysaccharide
**L4**	Fourth stage larvae
**LVFX**	Levofloxacin
**MABA**	Microplate Alamar Blue assay
**MTB**	*Mycobacterium tuberculosis*
**MDR**	Multidrug resistance
**MIC**	Minimum inhibitory concentration
**MSSA**	Methicillin-sensitive *S. aureus*
**MRSA**	Methicillin-resistant *S. aureus*
**MSSE**	Methicillin-sensitive *S. epidermidis*
**MXFX**	Moxifloxacin
**MMP**	Matrix metalloproteinase
**MCF-7**	Breast cancer cell line
**MDA-MB-231**	Human breast adenocarcinoma cell
**MTS**	3-(4,5-Dimethylthiazol-2-yl)-5-(3-carboxymethoxyphenyl)-2- (4 sulfophenyl)-2H-tetrazolium
**MES**	Maximal electroshock
**MV4-11**	Acute myeloid leukemia
**MiaPaCa**	Pancreas cancer
**MRC-5**	Human fetal lung fibroblast cells
**NO**	Nitric oxide
**NIH/3T3**	Non-cancerous murine embryonal fibroblasts
**NAAA**	N-Acylethanolamine acid amidase
**NMDA**	N-Methyl-D-aspartic acid
**PEA**	Palmitoylethanolamide
**Pc-3**	Prostate cancer
**QSAR**	Quantitative structure–activity relationship
**ROS**	Reactive oxygen species
**SAR**	Structure–activity relationship
**SH-SY5Y**	Neuroblastoma cell
**SW480**	Colon cancer
**scPTZ**	Subcutaneous pentylenetetrazole
**TB**	Tuberculosis
**TR-146**	Human squamous cell carcinoma
**XTT**	2,3-Bis-(2-methoxy-4-nitro-5-sulfophenyl)-2H- tetrazolium-5-carboxanilide
**α-Gal A**	α-Galactosidase A
**3OGN**	Crystal structure of an odorant-binding protein
**6 Hz**	Psychomotor tests
